# Victimization and Perpetration of Sexual Violence in College Men: The Characteristics of Social Ecology, Shared Risk Factors and Their Implications for Prevention

**DOI:** 10.1080/10926771.2025.2549743

**Published:** 2025-08-30

**Authors:** Jasmine A. Skorheim, RaeAnn E. Anderson

**Affiliations:** a Department of Psychology, University of North Dakota, Grand Forks, ND, USA;; b Department of Psychological Science, Kent State University, Kent, OH, USA;; c Department of Health Sciences, University of Missouri – Kansas City, MO, USA

**Keywords:** College men, impulsivity, interpersonal relationships, interpersonal skills, sexual assault, sexual violence, sexual violence prevention, social norms

## Abstract

Previous research has demonstrated a relationship between victimization and perpetration of sexual violence. Social norms approaches have been developed to prevent sexual violence, which focus on changing perceived peer support for sexual violence and related behaviors; however, their efficacy is limited. Interpersonal difficulties are a well-established consequence of sexual victimization much like impulsivity is a well-established risk factor for sexual perpetration, however, these risk factors are often considered in relation to one form of violence rather than both. The goals of this study were to 1) describe and assess the relationship between knowledge of peers’ sexual violence experiences and 2) examine the afore-mentioned risk factors while considering victim-offender overlaps to inform prevention and restorative justice efforts. College men (*n* = 485) completed an anonymous online survey. 46.4% reported sexual violence exposure, specifically 10.5% victimization only, 6% perpetration only, and 29.9% both exposures. Many participants reported knowing a victim (43.9%) or perpetrator (32.4%), mostly consisting of friends and acquaintances. Violence-exposed groups demonstrated significantly more knowledge of victimization (Victimization Only 52.9%, Perpetration Only 62.1%, Both 61.4% > Control 30.4%) and perpetration (Perpetration only 55.2%, Both 49% > Control 20.8%) amongst their peers than nonviolence-exposed controls. Those with victimization only reported greater interpersonal difficulties than nonviolence-exposed controls (*d* = 0.613). When accounting for childhood sexual abuse (CSA), those with all three experiences had greater interpersonal difficulties (*d* = 0.766). Those with perpetration experiences demonstrated greater levels of impulsivity than nonviolence-exposed controls (*d* = 0.440). When accounting for CSA, those with both exposures (excluding CSA) had greater impulsivity (*d* = 0.488).

Epidemiological data suggest that approximately one-third of Americans experience victimization of sexual contact (Basile et al., 2022). Sexual victimization is any experience of sexual activity that occurs without consent and is most often perpetrated by someone known to the person, such as a friend, romantic partner, or acquaintance (Basile et al., 2022). Considering healthy adults, much less is known about those who cause this harm; in other words, the etiology of sexual perpetration. To date, there have been no successful, nationally representative epidemiological studies to point toward the population prevalence of sexual perpetration (Stockman et al., 2023). This is in contrast to related fields such as justice-involved individuals with sexual offending histories and research on sexual victimization in college and community women. Systematic reviews suggest approximately one-third of college men perpetrate contact sexual violence of some type, but their acknowledgment or awareness of this behavior is incredibly low, with approximately 1% of college men acknowledging their own perpetration behavior (Anderson et al., 2021). Further, intervention to prevent perpetration is difficult with well-documented boomerang effects (i.e., result in the opposite reaction than intended), including defensive reactance induced by programs focused on attitude change, which can actually worsen outcomes (Malamuth et al., 2018). Finally, to the authors’ knowledge, there are no established sexual victimization risk-reduction programs designed for cisgender men, as many sexual violence prevention programs directed at men focus only on perpetration (Orchowski et al., 2020). The overall goal of the current studies was to examine the overlap between sexual victimization and perpetration in college men. The goal of Study 1 was to examine the social ecology of college men, particularly their relationships with peers and knowledge of their peers’ exposure to sexual violence, to better inform social-ecology theory-based interventions. The goal of Study 2 was to examine whether well-established, modifiable risk factors (interpersonal skills and impulsiveness) that have been associated with specific individual forms of violence (victimization *or* perpetration) in prior research are in fact shared risk factors across forms of violence (victimization *and* perpetration).

## Victim–offender overlap

One potentially overlooked and complicated factor in perpetration intervention is prior histories of victimization. The relationship between victimization and perpetration of offenses is often referred to as victim-offender overlap, as many perpetrators of offenses tend to report prior victimization and vice versa (Berg & Mulford, 2020; Jennings et al., 2012). Perpetrators with victimization histories are sometimes referred to as “complex victims,” as they face unique and multifaceted barriers to justice and treatment (Weiss, 2022). While this phenomenon has been studied generally, Miley et al. (2020) evidenced that the victim-offender overlap should be viewed in terms of analogous acts (e.g., sexual victimization to sexual perpetration) rather than generally (i.e., *any* form of victimization leading to *any* form of perpetration). The cycle of violence theory (Widom, 1989a, 1989b) has been applied to explain the victim-offender overlap stemming from childhood victimization. Studies have demonstrated support for the cycle of violence in sexual violence, finding that childhood sexual abuse (CSA) is significantly associated with the perpetration of sexual violence in adulthood in samples of incarcerated (Felson & Lane, 2009) and college men (Loh & Gidycz, 2006). CSA has also been found to be a significant predictor of revictimization of sexual violence in adolescence/adulthood (Krahe & Berger, 2017).

Recent research has extended this paradigm to more specifically examine the impact of victimization experiences occurring in adolescence and adulthood, not just childhood. Peterson et al. (2019) found significant overlap between perpetration and victimization of sexual violence in adult men, regardless of whether the victimization experience occurred in childhood or adolescence/adulthood, with 48.9% of victims reporting perpetration and nearly identical numbers vice versa. Leiding et al. (2021) found that adult men with prior exposure to sexual violence were seven times more likely to become sexual perpetrators than those without such exposure. This research suggests perpetration prevention programming may need to account for experiences of previous victimization. While a link between victimization and perpetration exists, it does not imply causation (i.e., not *all* victims become perpetrators and vice versa).

## Current prevention programming for college men

In one of the first systematic reviews on the topic, DeGue et al. (2014) found that many of the existing prevention programs for sexual violence perpetration were understudied, ineffective (i.e., consistent null findings), or even harmful, with only 3 interventions (2.1%) deemed effective. A more recent review by Wright et al. (2020) found that perpetration prevention programs that focus on the perpetrators themselves, rather than bystanders, do not effectively reduce perpetration rates, consistent with the boomerang effect. Since DeGue et al. (2014)’s review, the most promising interventions have been those with focus on bystander interventions rather than individual attitude change (Banyard et al., 2007; Coker et al., 2015; Gidycz et al., 2011; Kettrey & Marx, 2021). Coker et al. (2015) found that the Green Dot bystander intervention reduced victimization by 11% and perpetration by 19% on college campuses. A unique component of Green Dot, in comparison to other bystander interventions, is the training of influential peers to promote intervention strategies. This points to the power of interpersonal relationships to potentially prevent perpetration behavior. An evaluation of Coaching Boys Into Men showed decreased dating violence perpetration and increased positive bystander intervention (Miller et al., 2012), another example of leveraging interpersonal skills and relationships. However, Sorenson et al. (2014) point out a gap in the literature regarding people’s personal knowledge of and relationships to others with sexual violence experiences; knowledge of other victims/perpetrators could create ambivalence or motivate change toward prevention strategies.

Recent research has also suggested promise in restorative justice programs, which focus on relational factors for sexual violence perpetration, however, this data is limited (Burns & Sinko, 2023). Thus, new strategies and targets for prevention should be explored. Given the victim-offender overlap demonstrated in sexual violence, better understanding this overlap and the associated social ecology may be especially important while also avoiding the boomerang effect.

## Brief introduction study 1: Victim–offender overlap and social ecology

Social norms (i.e., the perceived attitudes and behaviors of others in interpersonal environments: Berkowitz et al., 2022) play an important role in understanding the causes of sexually violent behaviors, especially in theory-driven work using the social-ecological model (i.e., the interaction between individual, relational, communal, and societal factors: Centers for Disease Control and Prevention, 2024b). In addition to understanding how previous exposure to sexual violence may impact future risk, understanding relationships with other victims and perpetrators, or personal knowledge of them, may impact future risk (Sorenson et al., 2014). For example, situational crime prevention theory emphasizes changing environmental cues that prompt criminal behavior, exert social pressure on offenders, or weaken moral constraints against offending (Wortley & Smallbone, 2006). In the case of college men and sexual perpetration, social norms around how sexual advances should be made, how to communicate sexual desires, and perhaps most importantly, when to stop advances are all important parts of the individual, relational, communal, and societal levels of the social ecology. The more active men are in their social groups (Kaczkowski et al., 2017), and the more diverse and denser those social groups are (Kaczkowski et al., 2017; Swartout, 2013), the less likely they are to perpetrate sexual violence. Additionally, men who perpetrate sexual violence are more likely to overestimate their friends’ support for and engagement in sexually violent behavior (Dardis et al., 2016), and perceived peer approval is a risk factor for sexual violence perpetration (Swartout, 2013).

Social support in close relationships is associated with improved recovery after victimization (Dworkin et al., 2018) and better wellbeing and functioning (Hamby et al., 2020). One recent study found that social connectedness was protective against attitudes associated with risk for sexual perpetration (O’Connor et al., 2024), suggesting that enhancing social relationships may not just be a pathway to resilience after victimization, but also a strength to prevent violence. Therefore, it is important to better understand the social ecology of sexual violence, particularly relational and interpersonal factors, as social-ecological factors have important implications for both skill-based (e.g., building relational skills) and social norms-based (e.g., changing ideas around what is normal dating behavior) prevention programming. Social-ecological factors could be especially useful in restorative justice approaches, which center and leverage personal relationships (Burns & Sinko, 2023; Orchowski et al., 2020).

### Study 1: The current study

Study 1 examined the relationship between the characteristics of young men’s relationships and their own sexual violence histories to inform social norms approaches to sexual violence risk-reduction and prevention efforts for men. This was assessed by comparing groups of participants with experiences of victimization only, perpetration only, and both victimization and perpetration of sexual violence, replicating Peterson et al. (2019) approach. Sexual victimization was considered across the lifespan, including experiences in childhood and adolescence/adulthood, rather than focusing on only one developmental period. The first aim of the current study was to describe the number of participants who reported knowledge of peers’ experiences of sexual violence, revisiting a phenomenon rarely described since Sorenson et al. (2014). Further, it was hypothesized that 1) each violence-exposed group would have more knowledge of peers with similar experiences (victimization, perpetration, or both) compared to nonviolence-exposed controls (Sorenson et al., 2014). The current study also examined social ecological characteristics of the groups, hypothesizing 2) that those with perpetration histories (Perpetration Only and Both groups) would demonstrate less diversity and closeness in their relationships (Dardis et al., 2016; Kaczkowski et al., 2017; Swartout, 2013). The current study also sought to better characterize and expand how diversity in relationships is considered by inquiring about the gender and sexual identities of reported friends compared to prior research (Kaczkowski et al., 2017).

### Study 1: Method

#### Participants and procedure

College men (*N* = 485) were recruited online through Kent State University’s Sona Systems for a parent study on labeling peer perpetration behavior (Anderson et al., 2017) (see Supplemental Materials for more information). Though perpetration of sexual violence is not exclusively perpetrated by men (Stemple et al., 2017), the current study recruited college men given available measures of sexual violence perpetration may not be valid for college women (Buday & Peterson, 2015). Of note, participants were recruited from psychology courses and received psychology course credit for participating, however, only 16.7% of participants were psychology majors. Participants ranged from 18 to 44 years old (*M* = 19.77, *SD* = 2.36). Participants selected all race options applicable to them, with 80.4% White, 9.1% African American, 5.2% Asian American, 3.7% Hispanic/Latino, 1.4% Native American/American Indian, and 5.4% Other. Most participants identified as heterosexual (85.8%) and others identified as a sexual minority (11.5%), including bisexual (5.4%), gay (4.9%), and other (1.2%).

Participants completed a total of 15 questionnaires, with each questionnaire containing 5–39 items, as part of the larger parent study. Study questionnaires were presented to participants in a randomized order. Seven of these questionnaires were utilized in Study 1, two additional questionnaires are presented in Study 2, and six other questionnaires were administered but not included in this paper (see Supplemental Materials for more information). All study procedures were completed at one point in time; findings are presented as Study 1 and Study 2 based on conceptual similarities in the questionnaire content and theory.

#### Measures

##### Childhood trauma questionnaire (CTQ).

The CTQ assesses childhood maltreatment using five subscales: physical abuse, physical neglect, emotional abuse, emotional neglect, and sexual abuse (Bernstein et al., 1994). This scale has evidence of strong internal consistency, with Cronbach’s α = 0.89 in this sample. Participants were asked to indicate how truly (never true, rarely true, sometimes true, often true, very true) each item from the physical, emotional, and sexual abuse subscales (15 items total) described their experiences prior to the age of 18. The sexual abuse subscale was utilized to assess CSA.

##### Sexual experiences survey – Short form victimization (SES-SFV) and perpetration (SES-SFP).

The SES-SFV consists of five items for men in which sexual victimization history is assessed by presenting the participant with a specific sexual act (e.g., “someone fondled, kissed, or rubbed up against the private areas of my body”) and providing several different options of coercive tactics that may have been used to make them engage in that sexual act (e.g., “telling lies, threatening to end the relationship, … or continually pressuring after I said I didn’t want to”) (Koss et al., 2007). This measure has evidence of adequate convergent validity and test–retest reliability in college men (Anderson et al., 2018). The items for men exclude questions involving vaginal penetration. Participants were instructed to indicate the number of times (0–99) they experienced each item in a fill in the blank box. When it was observed that this response format was suppressing responding for the first 125 participants (5.0% vs. 17.9% victimization rate; χ^2^(1, *N* = 485) = 20.232, *p* < .001*), the format was switched to the standard format (0,1,2,3+ times). The SES-SFP consists of seven items (including vaginal penetration) mirroring the SES-SFV. Participants were given the same response format as for the SES-SFV and similarly, endorsement rates increased when the format was switched to the standard format (2.5% vs 7.3%; χ^2^(1, *N* = 485) = 5.995, *p* = .014*).

##### Conflict tactics Scale (CTS2).

The CTS2 consists of 39 items assessing violence in intimate relationships through the following subscales: physical assault, psychological aggression, negotiation, injury, and sexual coercion (Straus et al., 1996). Participants were presented with each item (e.g. “I used force … to make my partner have sex”) and were then prompted to indicate if “my partner did this to me.” Participants were instructed to indicate how many times (never, once, twice, 3–5 times, 6–10 times, 11–20 times, more than 20 times) their partner perpetrated each behavior against them in the past year and anytime since the age of 14 (not in the past year). The same items were utilized to assess perpetration, asking participants to indicate how many times they themselves had engaged in each behavior. The seven-item sexual coercion subscale was utilized to assess participants’ sexual violence history with intimate partners.

##### Victimization and perpetration knowledge.

Personal knowledge of sexual violence victimization was assessed using an adapted version of Sorenson et al. (2014) victimization knowledge questions. This measure consists of eight items prompting yes or no responses to questions about victimization scenarios that someone they know has experienced (e.g., “Do you know anyone who ever has given in to sex play … when she didn’t want to because she was overwhelmed by a man’s continual arguments and pressure?”). If participants indicated yes to any of these items, they were asked about their relationship to the person who experienced it. Participants were able to indicate multiple different types of relationships to victims. The eight-item Perpetration Knowledge Questionnaire mirrored these items. The full questionnaires are provided in Supplemental Materials. This measure has evidence of good internal consistency, with Cronbach’s α = 0.901 for the victimization knowledge questions and Cronbach’s α = 0.838 for the perpetration knowledge questions in this sample.

##### Relationship characteristics.

Existing measures of interpersonal closeness all focus on singular relationships (i.e., think about your best friend) rather than closeness across the range of relationships. Prior research has only considered relationship diversity by domain of social activity (e.g., friend, work, school: Kaczkowski et al., 2017) whereas this study considered diversity in terms of gender and sexual identity. Therefore, the authors created a brief questionnaire for this study consisting of 14 items which assessed individuals’ interpersonal relationships and the characteristics of these relationships. The full questionnaire can be found here: https://osf.io/6xr3j/?view_only=859b58d125b34a5d8d39b1a2fb667aed. Items consisted of statements like, “Do you have a best or very good friend, a friend you feel very close to you? By feel close we mean a friend you feel you can share personal information with or discuss problems in your life with,” to measure closeness and statements like, “Do you have any friends that are not the same sexual orientation as you? Yes, maybe/unsure, no,” to assess diversity in relationships. When participants provided a range of numbers in response to the open-ended items like “How many close friends do you have that are men?,” the average value was utilized in scoring. Participants responses to open-ended items that did not include numerical values (“a lot,” “many,”) were excluded. Participants also responded to items measuring feelings of responsibility for violence (see Supplementary Materials for more details and results).

#### Analytic plan

Group differences were measured by one-way ANOVAs with follow-up Dunnett T3 tests (to account for unequal variance based on dissimilar group sizes) to specify between group differences for continuous variables. For ratio variables, chi-square tests were used. Fisher’s exact tests were used when cell sizes were <10. Social desirability did not have a confounding effect on results (see Supplemental Materials for more details).

### Study 1: Results

#### Descriptive statistics

Almost half of the sample (*n* = 225; 46.4%) reported experiences of sexual violence of some type. 10.5% (*n* = 51) reported experiences of victimization only, 6.0% (*n* = 29) reported experiences of perpetration only, and 29.9% (*n* = 145) reported experiences of both victimization and perpetration. Prevalence rates for CSA and related descriptive statistics are available in Supplemental Materials. 43.9% of the sample reported knowledge of victimization in peers; 32.4% reported any knowledge of perpetration in peers.

#### Aim and hypothesis 1: Knowledge of victimization in peers

**Figure 1.**
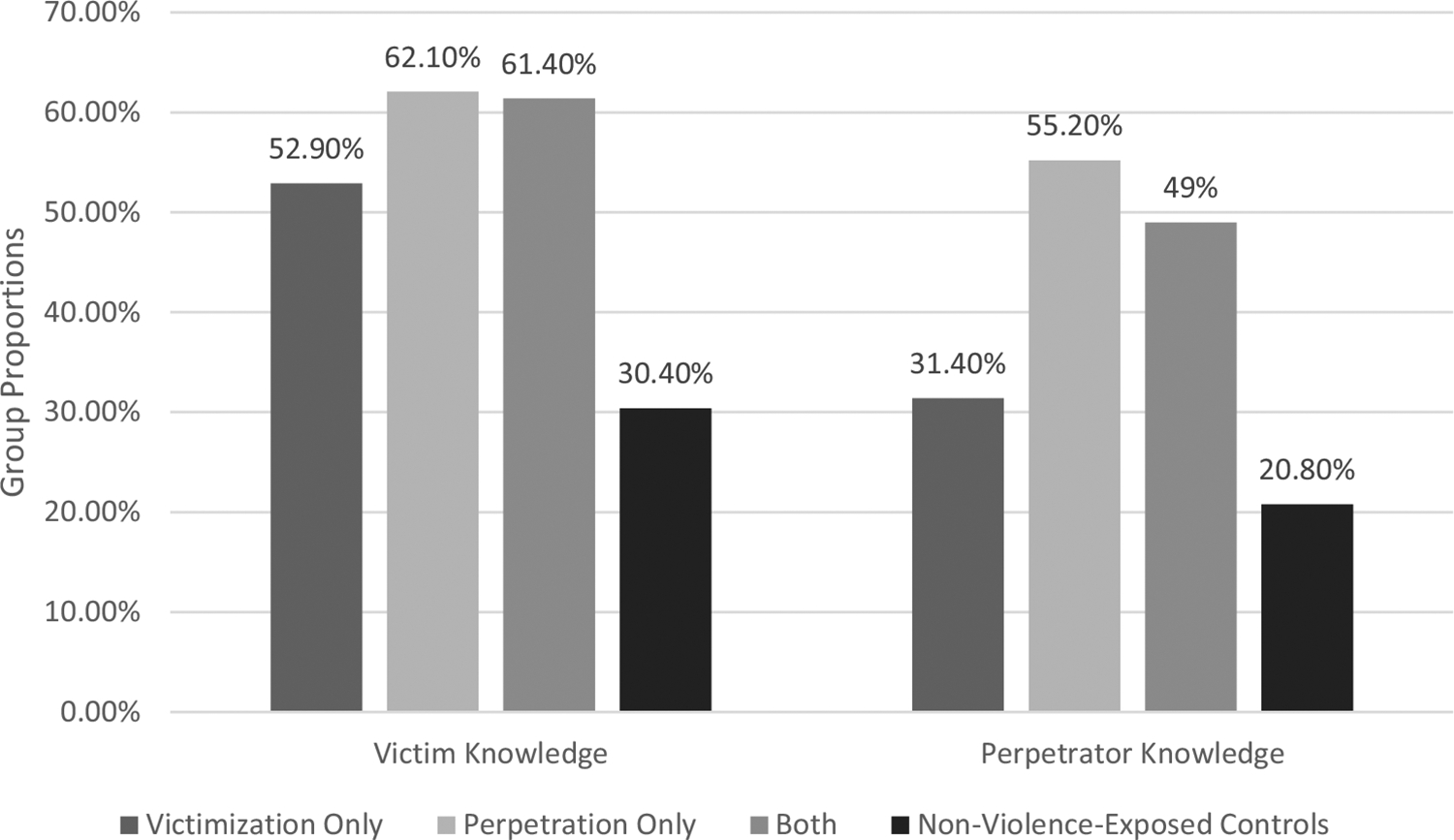
Study 1: knowledge of peers with sexual violence exposure. *Note*. Victimization knowledge (χ^2^(3, *N* = 485) = 42.849, *p* < .001*, Cramer’s *V* = .297, Cohen’s *d* = .623) and perpetration knowledge (χ^2^(3, *N* = 485) = 41.135, *p* < .001*, Cramer’s *V* = .291, *d* = .609) were not distributed evenly between groups.

**Figure 2.**
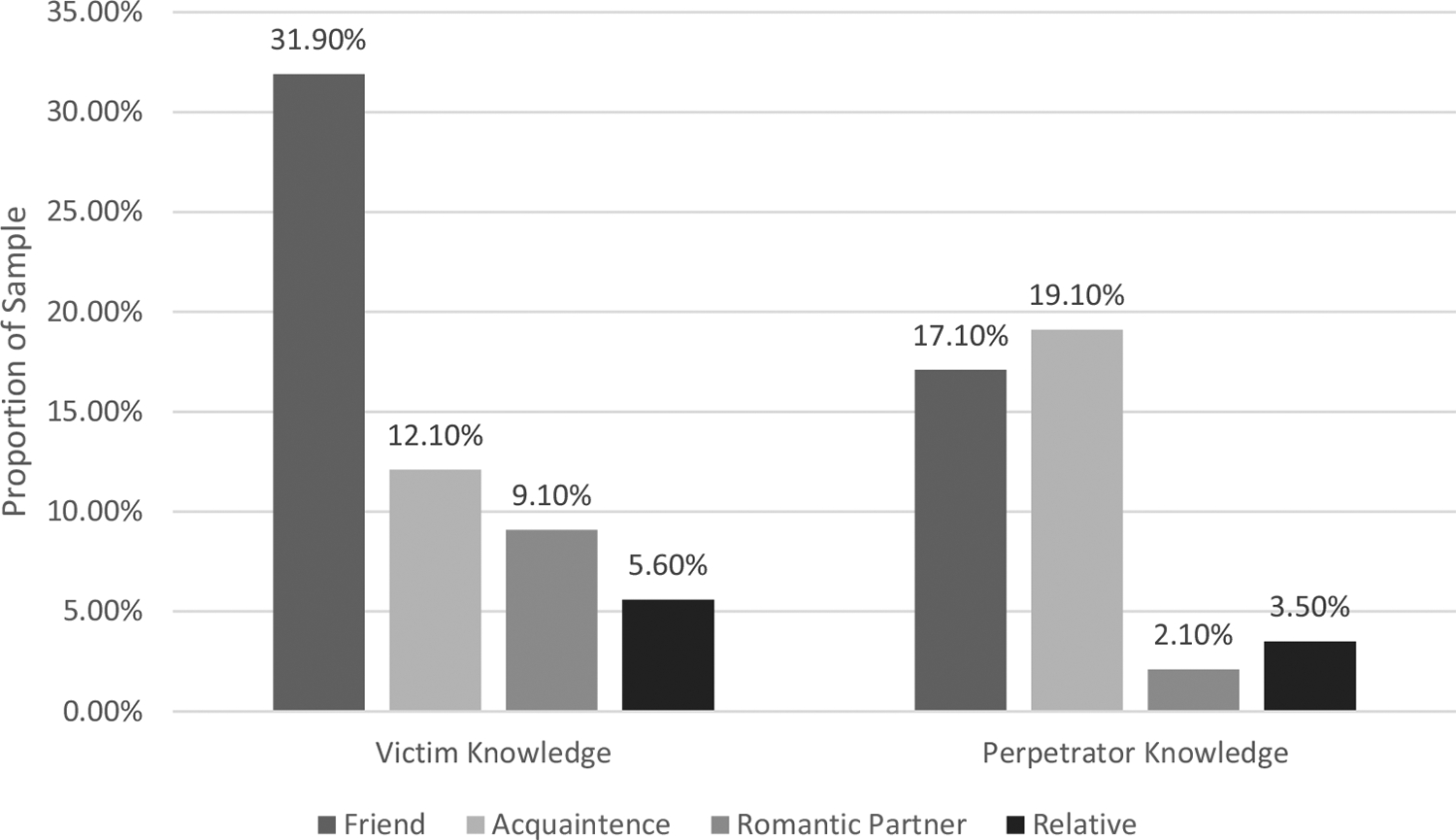
Study 1: relationships to peers with sexual violence exposure.

It was predicted that each group would have more knowledge of peers with similar experiences (victimization, perpetration, or both) compared to nonviolence-exposed controls. Overall, almost half of the sample (43.9%) reported victimization knowledge (see Figure 1). More specifically, 52.9% of the Victimization Only group, 62.1% of the Perpetration Only group, and 61.4% of the Both group reported victimization knowledge in comparison to 30.4% of nonviolence-exposed controls. Follow-up tests indicated a significantly greater proportion of the Victimization Only group (χ^2^(1, *n* = 311) = 9.656, *p* = .002*, Cramer’s *V* = .176, *d* = .358), Perpetration Only group (χ^2^(1, *n* = 289) = 11.746, *p* < .001*, Cramer’s *V* = .202, *d* = .412) and the Both group (χ^2^(1, *n* = 405) = 36.839, *p* < .001*, Cramer’s *V* = .302, *d* = .633) reported victimization knowledge than nonviolence-exposed controls (52.9%, 62.1%, 61.4% > 30.4%). There were no significant differences found between violence-exposed groups. Of those who reported victimization knowledge, 72.8% of those who reported their relationship to the peer was a friend, 27.7% acquaintance, 20.7% romantic partner, and 12.7% a relative (see Figure 2).

#### Aim and hypothesis 1: Knowledge of perpetration in peers

Almost one-third of the sample (32.4%) reported perpetration knowledge. More specifically, 31.4% of the Victimization Only group, 55.2% of the Perpetration Only group, 49% of the Both group, and 20.8% of nonviolence-exposed controls reported perpetration knowledge, which was not evenly distributed across groups (see Figure 1). A significantly greater proportion of the Perpetration Only group (χ^2^(1, *n* = 289) = 16.824, *p*= < .001*, Cramer’s *V* = .241, *d* = .497) and the Both group (χ^2^(1, *n* = 405) = 34.683, *p* < .001*, Cramer’s *V* = .293, *d* = .612) reported perpetration knowledge than the nonviolence-exposed controls (55.2%, 49% > 20.8%). Similarly, a significantly greater proportion of the Perpetration Only group (χ^2^(1, *n* = 80) = 4.639, *p* = .037*, Cramer’s *V* = .234, *d* = .480) and the Both group (χ^2^(1, *n* = 196) = 4.731, *p* = .030*, Cramer’s *V* = .155, *d* = .315) reported perpetration knowledge than the Victimization Only group (55.2%, 49% > 31.4%). Of those who reported perpetration knowledge, 59.2% of those who reported their relationship to the peer was an acquaintance, 52.9% friend, 10.8% relative, and 6.4% romantic partner (see Figure 2).

#### Hypothesis 2: Relationship characteristic findings

It was predicted that those with perpetration histories would demonstrate less diversity and closeness in their relationships. Groups did not differ in having a close friendship, the number of friendships, nor diversity in friendships (see Supplementary Materials for more details). Closeness in relationships was further assessed by examining emotional intimacy and disclosures (i.e., what type of emotional information participants reported being willing to share with friends) and did vary across groups (see Table 1). A significantly greater proportion of the Perpetration Only group and the Both group reported sharing whether they were having sex in a relationship with their friends than nonviolence-exposed controls. A greater proportion of the Victimization Only group and the Both group reported sharing anger/frustration in their relationship with their friends than nonviolence-exposed controls. There were also differences in sharing sadness and not sharing information (see Table 1).

#### Study 1: Brief discussion

Study 1 assessed the relationship between the overlap of victimization and perpetration and characteristics of the social ecology. *Many* college men reported knowing peers who had experienced victimization (43.9%) and perpetration (32.4%) (Aim 1). Consistent with hypotheses, violence-exposed individuals were more likely to know peers who had also been exposed to similar types of sexual violence (H1). Regarding the relationship to victimization-exposed peers, most were friends. In slight contrast, those who reported perpetration knowledge were equally likely to report the peer was an acquaintance or friend. These victimization findings were strikingly similar to Sorenson et al. (2014)’s epidemiological sample estimates. However, this sample reported less knowledge of perpetration (32.4% vs. 45%) than theirs.

Considering relationship characteristics, there were not significant differences between violence-exposed groups in the number or diversity of friendships, however, there were differences in the emotions individuals were willing to share with their friends (H2). A greater proportion of those with perpetration histories reported sharing information about having sex in their relationships than nonviolence-exposed controls. This is consistent with American norms around masculinity in that having sex is a status symbol to be shared, a phenomenon that has been associated with rape myth acceptance (Cole et al., 2020). This may be why those who perpetrate overestimate their friends’ support and frequency of perpetration behaviors – they tend to share this kind of information more often than other intimate information. Further, they are assuming their own experiences are the norm, and, to some degree, they are correct given that perpetration knowledge was more equally distributed between acquaintances and friends than victimization knowledge.

## Brief introduction study 2: Shared risk factors

Given the high overlap of victimization and perpetration documented in Study 1, the authors further investigated the potential implications of this overlap by focusing on risk factors demonstrated as modifiable in prior research and tied to skill-building interventions. While there are certainly likely other shared risk factors, there are no comprehensive literature reviews on risk factors for sexual victimization in men like there are for victimization in women (e.g., Muehlenhard et al., 2017) or perpetration in men (e.g., O’Connor et al., 2021) that could be consulted. Another deciding factor in selecting these risk factors is that interpersonal skills and impulsivity are logical extensions of the social ecological model; those with poorer interpersonal skills may be less likely to be disclosed to by friends and have difficulty in relationships.

### Interpersonal skills

Interpersonal skill is a broad construct including sub-constructs such as social skills, intimacy, interpretation of cues from others, social desirability, empathy, etc (Tharp et al., 2013). Those who experience childhood sexual abuse (CSA) have been reported to experience a variety of interpersonal difficulties (e.g., isolation, decreased trust, fewer relationships: Briere & Elliott, 1994), which can extend into adulthood (e.g., dissatisfaction in relationships, issues with intimacy/sexual functioning: Briere & Elliott, 1994; Davis & Petretic-Jackson, 2000; DiLillo, 2001). Further, a link between interpersonal skills difficulties and sexual violence perpetration has been demonstrated in samples of incarcerated (Friestad & Vaskinn, 2021) and college men (Carvalho & Sá, 2020; Loh & Gidycz, 2006). Given these findings, interpersonal skills difficulties caused by CSA may be an explanatory mechanism of the cycle of violence. Interpersonal skills difficulties appear to be a shared risk factor for adolescent and adult victimization regardless of CSA history, as interpersonal skills predict the ability to use self-defense (Anderson et al., 2020) and one of the few effective perpetration prevention programs includes conflict resolution training (Miller et al., 2020). Further, interpersonal skills are a modifiable risk factor which can be changed through skills-based intervention. Indeed, interpersonal skills could also be conceptualized as a protective factor, given interpersonal skills are associated with acquiring social support and other indicators of wellbeing (Segrin & Taylor, 2007).

### Impulsivity

Experiences of CSA and adolescent/adult victimization have been associated with multiple behavioral health outcomes driven at least in part by impulsivity, such as substance use and risky sexual behaviors (Kerig, 2019; Kilimnik et al., 2023; Testa et al., 2010). Further, these behavioral health outcomes are linked to later revictimization (Krahe & Berger, 2017), suggesting that these impulse-control related consequences of CSA are one potential mechanism of revictimization. Perpetration of sexual violence has similarly been linked to impulsivity (Tharp et al., 2013), with those who perpetrate acting on emotion during positive or negative urgency (Mouilso et al., 2013; Ybarra et al., 2023). In a strong causal design, a large longitudinal study of college men found impulsivity was one of three risk factors that mediated the impact of alcohol on sexual perpetration (Testa & Cleveland, 2017). Impulsivity appears that it may be a shared risk factor for violence involvement regardless of CSA history considering how impulsivity is related to substance use, which can impair risk recognition and response (Messman-Moore & Long, 2003; Verdejo-Garcia et al., 2008) and inhibition of harmful behaviors (Testa & Cleveland, 2017). Like interpersonal skills, through skills-based interventions, impulsivity can be modified and reduced, which, in turn, can reduce aggressive behaviors (Johnson et al., 2020).

### Study 2: The current study

Study 2 examined potentially shared risk factors while considering the victim-offender overlap in the same sample as Study 1. However, given the wealth of research on CSA and interpersonal skills, rather than defining victimization partially by CSA status, CSA was considered separately to provide clarity on potentially unique impacts of CSA. It was hypothesized that 1) all violence exposed-groups (Victimization Only, Perpetration Only, Both groups) would demonstrate difficulties in interpersonal relationships (Briere & Elliott, 1994; Carvalho & Sá, 2020; Davis & Petretic-Jackson, 2000; DiLillo, 2001; Friestad & Vaskinn, 2021; Loh & Gidycz, 2006) compared to nonviolence-exposed controls and 2) all violence-exposed groups would demonstrate greater impulsivity than nonviolence-exposed controls (Kerig, 2019; Kilimnik et al., 2023; Mouilso et al., 2013; Testa et al., 2010; Tharp et al., 2013; Ybarra et al., 2023). Exploratory analyses were conducted to examine how the group differences in H1 and H2 (four group analysis) may be modified by considering CSA (eight group analysis). This analysis was considered exploratory given prior research on the impact of CSA has rarely included men (Thomas & Kopen, 2023), and that some of the cell sizes were very small, precluding true hypothesis testing.

### Study 2: Method

#### Measures

##### Inventory of interpersonal problems (IIP-32).

The IIP-32 consists of 32 items to assess difficulties in interpersonal relationships using eight subscales (Barkham et al., 1996). Items are composed of “hard to be” (e.g. “It is hard for me to join in on groups”) and “too” much (e.g. “I fight with other people too much”) questions. This measure has evidence of internal consistency, with Cronbach’s α = 0.924 for the overall scale in this sample. Participants were presented with each item and instructed to indicate how much the statement applies to them (not at all, a little bit, moderately, quite a bit, extremely). Interpersonal difficulties were indicated by higher scores.

##### UPPS impulsive behavior Scale.

The UPPS consists of 45 items to assess impulsiveness with the following subscales: urgency, premeditation, perseverance, and sensation seeking (Whiteside & Lynam, 2001). This measure has evidence of good internal consistency, with Cronbach’s α = 0.860 in this sample. Participants were presented with each item (e.g., “I like to stop and think things over before I do them”) and instructed to indicate how much they agree (agree strongly, agree somewhat, disagree somewhat, disagree strongly). Impulsiveness was indicated by higher scores.

#### Analytic plan and power analysis

Study 2 utilized the same analytic plan as Study 1. Prior research suggests that interpersonal skills and perpetration would be associated in the *d* = .3 range (*r* = .34 in (Carvalho & Sá, 2020). Mouilso et al. (2013), also using the UPPS with a sample of college men, estimated the association between sexual perpetration and impulsivity at *d* = .58. Thus, *d* = .3 was used to conduct *a priori* power analysis after establishing the relative proportions of the victim-offender overlap groups. For groups of unequal sizes in a ratio of .59, .29., 08., and .07, power analysis suggested that *N* ≈ 490 participants would be needed for omnibus ANOVA tests at Power = 0.80 and α = 0.05 with the smallest group *n* = 34. Because this was very close to the overall sample size of 485 and smallest subgroup of 35, the analysis was considered adequately powered.

### Study 2: Results

#### Descriptive statistics

Group size and composition were very similar to Study 1, with groups only varying in a different *n* = 6 (Perpetration Only, Both) and *n* = 15 (Victimization Only, control). Almost half of the sample (*n* = 210, 43.3%) reported experiences of sexual violence in adolescence/adulthood. More specifically, 7.4% (*n* = 36) reported experiences of victimization only, 7.2% (*n* = 35) reported experiences of perpetration only, and 28.7% (*n* = 139) reported experiences of both victimization and perpetration.

#### Hypothesis 1: Interpersonal skills

It was predicted that all violence-exposed groups would demonstrate difficulties in interpersonal relationships compared to nonviolence-exposed controls. However, there was only one difference found between the Victimization Only group and Control group. Those in the Victimization Only group had significantly higher too caring subscale scores than nonviolence-exposed controls (see Table 2).

#### Hypothesis 2: Impulsivity

It was predicted that violence-exposed groups would demonstrate greater impulsivity than nonviolence-exposed controls. Hypotheses were partially supported, as total and urgency subscales scores were greater in the Perpetration Only group and the Both group compared to nonviolence-exposed controls (see Table 2).

### Study 2 exploratory results: Analysis with CSA

#### Descriptive statistics

Exploratory analyses were conducted to examine how CSA experiences may modify these relationships. With CSA considered in analysis, an additional 15 participants were considered violence-exposed (*n* = 225; 46.5%). See Tables 3 and 4 for group breakdowns including CSA. In the following sections, results are discussed in order of similarities to the original analysis, then new interpretations are discussed.

#### Revisiting hypothesis 1: Interpersonal skills

With CSA considered, significance was found for IIP-32 total scores, such that the Both (+CSA) group had significantly higher scores than the Control (no CSA) group and the Both (no CSA) group (see Table 3). Additionally, more subscale scores (5 this analysis vs. 1 without considering CSA) were significant. Thus, the inclusion of CSA clarified findings in highlighting additional interpersonal skill differences within the Both group (+CSA, no CSA: total scores, hard to be assertive, hard to be sociable, too dependent) and additional differences between the Both groups and Controls (hard to be assertive, hard to be sociable, hard to be involved, too dependent).

#### Revisiting hypothesis 2: Impulsivity

The differences between the Both (no CSA and +CSA) and control (no CSA) groups were replicated for total scores and urgency scores, and an additional difference for sensation seeking scores was identified with one exception to this pattern within the Both groups. The Both (no CSA) group had greater sensation seeking scores than the Both (+ CSA) group (see Table 4).

### Study 2: Brief discussion

Study 2 assessed possible shared and modifiable risk factors for sexual violence intervention while considering victim-offender overlap in college men. On a measure of interpersonal difficulties, those in the Victimization Only group demonstrated significantly greater interpersonal difficulties than nonviolence-exposed controls (H1). The impact of considering CSA replicated primary findings and clarified additional subscales in which interpersonal skills were impaired while highlighting new differences between the Both groups and nonviolence-exposed controls. Thus, college men who are exposed to any sexual violence (victimization or perpetration) struggle with interpersonal skills generally, even more so in specific areas if they also have a history of CSA, which is consistent with prior research on CSA (Briere & Elliott, 1994; Davis & Petretic-Jackson, 2000; DiLillo, 2001; Walker et al., 2005). These findings also complicate the literature, emphasizing that perpetration when co-occurring with victimization, not just victimization alone, is also associated with interpersonal skill difficulty. Regarding impulsivity, the Perpetration Only group and the Both group scored significantly higher on the total score and the urgency subscale measure of impulsivity than nonviolence-exposed controls (H2). With CSA considered, a similar pattern emerged as for interpersonal skills with one complicating finding for sensation seeking – the Both (no CSA) group had higher sensation seeking scores than the Both (+CSA) group, suggesting there may be something unique about impulsivity and violence involvement in the adolescent/adult years. This pattern, while surprising, makes sense given prior research likely recruited samples with high victim-offender overlap but did not know it; many studies only assess one construct and not the other, or assess only childhood sexual abuse for victimization.

## General discussion

The current study examined the victim-offender overlap, characteristics of the social ecology, and shared psychological risk factors for sexual violence victimization and perpetration in college men to highlight unique considerations for intervention. The current study found that, at least in this sample, victim-offender overlap is the norm, not the exception for violence-involved college men. To wit, the number of men who met criteria for both victimization and perpetration was approximately 4x larger than those who met only the victimization or perpetration group criteria. Though representative data on sexual violence perpetration are limited given perpetration is rarely included in epidemiological research, it is estimated that approximately one-third of college men perpetrate sexual violence (Anderson et al., 2021) and one-third of men experience sexual violence victimization involving physical contact in their lifetime (Centers for Disease Control and Prevention, 2024a). These estimates suggest the current study may reflect college men’s experiences of sexual violence, however, because perpetration and victimization were not measured simultaneously in these samples, the representativeness of these findings cannot be determined with certainty. Further, it is common for college men to know friends and acquaintances who have been victimized (43.9%) or have perpetrated (32.4%). When group composition was reexamined to account for CSA, all differences in risk factors between only victimization and perpetration groups disappeared and were better characterized as differences between the victim-offender overlap group and nonviolence-exposed controls. In sum, this research suggests interventions to curb sexual violence in college men must consider victim-offender overlap, knowledge of these behaviors in the social ecology, and shared pathways to violence involvement rather than focusing on only victimization or perpetration.

### Implications

These findings emphasize the frequency of the victim-offender overlap, as 73.8% of victims reported perpetration and 85.6% of offenders reported victimization, suggesting that studying either phenomenon alone in college men may produce misleading findings. Prevention and intervention efforts should consider how learning about sexual violence may elicit new understandings and emotional reactions of participants’ prior experiences, using a trauma-informed approach (Han et al., 2021; McCauley & Casler, 2016). Interventions should continue to challenge ideas that sexual violence is normal while helping participants process that information so that the feelings elicited about their own and peers’ experiences do not interfere with program delivery, but instead, enhance it. If the interventions do not account for these experiences and knowledge, the intervention may be perceived as irrelevant. Acknowledging and processing victim-offender overlap may be particularly helpful for restorative justice interventions; by being able to see themselves on “both sides” of the table, college men may be more willing or able to engage. Restorative justice and/or trauma-informed approaches that acknowledge the victim-offender overlap may also defuse common defensive reactions (e.g., Malamuth et al., 2018). Even if participants do not label their own experiences as victimization or perpetration; the high likelihood of knowledge of friends and peers’ experiences also supports restorative justice and/or trauma-informed approaches that allows college men to see sexual violence as a problem that affects them and their loved ones without activating defensiveness.

Prevention efforts could also use knowledge of violence in peers to activate men’s investment in these friendships, and therefore the health of their friends, to motivate behavior change. The general lack of difference in emotional disclosures to friends between those with and without perpetration histories suggest that those with perpetration histories likely have healthy social networks and could be recruited for interventions through social groups. Men who had been victimized were more likely to share certain vulnerable emotions, a potential strength that could be built upon for intervention. Group or dyadic-based interventions may be especially helpful for restorative justice interventions for those with perpetration histories, providing a built-in resource that can be motivating and help strengthen skills and facilitate repair. Finally, men with perpetration histories were more likely to report sharing information about their sex lives with friends; this suggests creating interventions targeted toward improving sexuality or sexual health outcomes may be appealing.

It is likely that the same mechanisms that lead to college men being more likely to experience victimization and perpetration rather than one or the other, knowing many friends who have also experienced violence, and being more willing to discuss sex in friendships is related to their poorer interpersonal skills and greater impulsivity. Considering a developmental trajectory, early intervention targeted toward healthy friendships and sex education may be beneficial. Even during college, offering interventions that address these factors in an approachable, value-congruent manner is likely worth-while. Considering the association between interpersonal skills and impulsivity with victim-offender overlap in sexual violence, prevention efforts should utilize skill-based interventions to address these shared risk factors. As Orchowski et al. (2020) suggested, perhaps the most effective prevention technique is a combination of skill-based interventions with bystander interventions to address the complex issues of individual risk factors in addition to the social norms associated with sexual violence. It is likely that the pathway to violence involvement for college men is littered with the same risk factors for victimization and perpetration until a certain point; it may only be that differences emerge in later, more proximal environments or conditions.

### Limitations and future directions

First, although data was collected regarding sexual violence experiences in childhood (0–14 years of age), since the age of 14, and in the past year (see Supplementary Materials for analysis of experiences since the age of 14 and in the past year between groups), the timeline of victim-offender overlap was not specifically assessed. Nor were the authors able to assess problematic sexual behaviors (e.g., precursors to perpetration) that occurred before age 14. Given the cycle of violence theory focuses on childhood abuse translating into adult perpetration (Widom, 1989a; 1989b), more clearly explicating timing would be fruitful in future research. Second, when assessing victim and perpetrator knowledge, items were constructed in such a way to only measure knowledge of female victims and male perpetrators, undercounting knowledge. Future research should further examine the psychometric functioning of this measure and revise it to be more comprehensive. Future research should assess the feelings/attitudes that people associate with this knowledge, as the current study does not assess whether participants recognized these experiences as sexual violence and how they felt about this knowledge. Third, in comparison to Peterson et al. (2019), the Both group was much larger, and has several possible explanations. It could be college men are uniquely violence-exposed in comparison to the community men in Peterson et al. (2019), something unique about this particular sample of college men, or that the measurement approaches used in this study were more effective in detecting violence. Thus, these findings of such high overlap in a relatively healthy sample demand replication, particularly in additional social and demographic groups. It may be that this degree of victim-offender overlap, knowledge of victimization/perpetration, and these shared risk factors are somewhat unique features specific to White, straight, male college student environments. Thus, investigating these relationships in other groups and understanding what environmental and cultural factors may facilitate or hinder these relationships is important. Finally, though the numbers are unexpectedly high, the relationship identified between victimization and perpetration does not imply causation, thus further research is needed on the protective factors in the cycle of violence and the victim-offender overlap.

### Conclusion

These studies demonstrated a very high amount of victim-offender overlap of sexual violence in a sample of college men with the Both group accounting for almost 30% of the sample rather than 6–10.5% for only perpetration or victimization. Many college men reported personally knowing someone who had been victimized (43.9%) or perpetrated sexual violence (32.4%). Considering this knowledge, plus their own sexual violence exposures, sexual violence intervention efforts for men should acknowledge these past experiences, help participants make sense of them, and build upon relationship-related strengths to reduce the occurrence and impact of sexual violence. This study also demonstrated that risk factors previously only associated with victimization or perpetration (interpersonal skills and impulsivity) were in fact better characterized as being associated with the victim-offender overlap. To address these shared risk factors, combining skill-based and bystander intervention techniques, as well as restorative justice and trauma-informed frameworks may be more efficacious than current approaches.

## Supplementary Material

Supp 1

Supplemental data for this article can be accessed online at https://doi.org/10.1080/10926771.2025.2549743

## Figures and Tables

**Table 1.** Study 1: Information/emotions shared with friends about romantic relationships between groups.

Information/Emotions Shared	Victimization Only (*n* = 51)	Perpetration Only (*n* = 29)	Both (*n* = 145)	Non-Violence-Exposed Controls (*n* = 260)	χ^2^ (1, *N* = 485)	*p*	Cramer’s *V*	*d*
Talks to friends about romantic relationships	78%	82.8%	84.1%	78.5%	2.116	.549	.067	.132
Shares problems/conflicts	84%	79.3%	80%	86.6%	3.451	.327	.086	.169
Shares if having sex in a relationship with friends	66%	82.8%	73.8%	55.6%	18.078	< .001*	.197	.394
	Perpetration Only > Control, Fisher exact *p* = .005*, Cramer’s *V* = .170, *d* = .335 Both > Control, χ^2^ = 12.870, *p* < .001*, Cramer’s *V* = .182, *d* = .362							
Anger or Frustration in relationship	70.6%	69%	61.4%	50%	11.831	.008*	.156	.316
	Victimization Only > Control, χ^2^ = 7.262, *p* = .007*, Cramer’s *V* = .153, *d* = .309 Both > Control, χ^2^ = 4.854, *p* = .028*, Cramer’s *V* = .109, *d* = .220							
Sadness or Disappointment in relationship	72.5%	69%	57.9%	51.2%	10.322	.016*	.146	.295
	Victimization Only > Control, χ^2^ = 7.875, *p* = .005*, Cramer’s *V* = .159, *d* = .322							
Love in relationship	58.8%	55.2%	60.7%	48.8%	5.876	.118	.110	.222
Happiness in relationship	72.5%	72.4%	70.3%	61.2%	5.419	.144	.106	.213
Does not share emotional information	17.6%	6.9%	13.1%	22.3%	7.994	.046*	.128	.259
	Control > Both, χ2 = 5.122, *p* = .024*, Cramer’s *V* = .112, *d* = .226							

**Table 2.** Study 2: risk factors between groups.

	Group Mean											
	Victimization Only (*n* = 36)		Perpetration Only (*n* = 35)		Both (*n* = 139)		Non-Violence-Exposed Controls (*n* = 275)					
Risk Factor	*m*	*SD*	*m*	*SD*	*m*	*SD*	*m*	*SD*	*F* (3, 482)	*p*	η2	*d*
**IIP-32 Scale**												
Total	1.73	0.56	1.68	0.65	1.53	0.59	1.46	0.61	3.216	.023*	.020	.286
	No significant differences detected between groups using Dunnett’s T3 follow-up tests											
Hard to be assertive	1.26	0.94	1.28	0.95	1.17	0.88	1.18	0.90	0.212	.888	.001	.063
Hard to be sociable	1.41	0.92	1.44	1.00	1.08	0.87	1.12	0.91	2.431	.064	.015	.247
Hard to be supportive	0.84	0.88	0.95	0.76	0.93	0.92	0.77	0.88	1.223	.301	.008	.180
Hard to be involved	1.39	1.01	1.41	0.94	1.30	0.92	1.09	0.96	2.603	.051	.016	.255
Too caring	2.48	0.89	2.14	0.91	2.10	1.16	1.96	0.85	3.421	.017*	.021	.293
	Victimization Only > Control, *p* = .012*, *d* = 0.613											
Too dependent	2.39	0.91	2.35	0.92	2.13	0.79	2.08	0.87	2.155	.093	.014	.238
Too aggressive	2.29	1.09	2.21	0.98	1.95	0.79	1.79	0.79	5.899	<.001*	.037	.392
	No significant differences detected between groups using Dunnett’s T3 follow-up tests											
Too open	2.01	0.96	2.35	0.63	2.20	0.74	2.08	0.75	2.099	.100	.013	.230
**UPPS Scale**												
Total	106.51	17.34	110.2	13.26	107.99	12.03	102.35	13.66	7.508	<.001*	.046	.440
	Perpetration Only > Control, *p* = .012*, *d* = .583; Both > Control, *p* < .001*, *d* = .438											
Premeditation	20.51	5.00	21.37	6.41	20.96	5.27	20.44	5.42	0.495	.686	.003	.110
Urgency	29.97	8.16	31.69	6.70	30.41	7.00	27.28	7.68	7.798	<.001*	.048	.450
	Perpetration Only > Control, *p* = .005*, *d* = .611; Both > Control, *p* < .001*, *d* = .426											
Sensation Seeking	36.51	6.40	37.11	7.28	37.12	6.56	35.46	6.93	2.083	.102	.013	.230
Perseverance	19.51	4.52	20.03	5.50	19.53	4.20	19.17	4.79	0.451	.716	.003	.110

**Table 3.** Study 2: IIP-32 scores between exploratory groups.

	Group Mean																			
	Victimization Only				Perpetration Only				Both				Non-Violence-Exposed Controls							
	No CSA (*n* = 25)		+CSA (*n* = 11)		No CSA (*n* = 29)		+CSA (*n* = 6)		No CSA (*n* = 104)		+CSA (*n* = 35)		No CSA (*n* = 260)		+CSA (*n* = 15)					
IIP-32 Scale	*m*	*SD*	*m*	*SD*	*m*	*SD*	*m*	*SD*	*m*	*SD*	*m*	*SD*	*m*	*SD*	*m*	*SD*	*F* (7, 478)	*p*	η2	*d*
Total	1.66	0.55	1.89	0.59	1.61	0.64	2.04	0.66	1.42	0.56	1.89	0.57	1.44	0.60	1.71	0.65	4.723	<.001*	.067	.536
	Both (+CSA) > Control (no CSA), *p* = .003*, *d* = .766; Both (+CSA) > Both (no CSA), *p* = .002*, *d* = .848																			
Hard to be assertive	1.16	0.99	1.48	0.80	1.24	0.95	1.46	1.04	0.98	0.82	1.75	0.83	1.17	0.90	1.37	0.89	3.082	.003*	.044	.429
	Both (+CSA) > Control (no CSA), *p* = .013*, *d* = .667; Both (+CSA) > Both (no CSA), *p* < .001*, *d* = .926																			
Hard to be sociable	1.42	0.95	1.39	0.90	1.29	0.96	2.13	0.96	0.89	0.83	1.66	0.76	1.11	0.91	1.38	0.82	4.737	<.001*	.067	.536
	Both (+CSA) > Control (no CSA), *p* = .008*, *d* = .656; Both (+CSA) > Both (no CSA), *p* < .001*, *d* = .969																			
Hard to be supportive	0.72	0.75	1.19	1.07	0.89	0.74	1.25	0.85	0.85	0.95	1.16	0.82	0.75	0.85	1.17	1.22	1.783	.089	.026	.327
Hard to be involved	1.27	1.03	1.64	0.97	1.32	0.92	1.88	0.96	1.19	0.95	1.60	0.79	1.08	0.97	1.30	0.92	2.329	.024*	.034	.375
	Both (+CSA) > Control (no CSA), *p* = .024*, *d* = .589																			
Too caring	2.39	0.88	2.68	0.91	2.07	0.95	2.46	0.66	2.04	1.20	2.31	1.00	1.94	0.84	2.20	0.97	2.145	.038*	.031	.358
	No significant differences detected between groups in follow-up analysis																			
Too dependent	2.28	0.78	2.61	1.15	2.35	0.95	2.38	0.88	2.00	0.77	2.52	0.71	2.05	0.86	2.50	0.92	3.091	.003*	.045	.434
	Both (+CSA) > Control (no CSA), *p* = .024*, *d* = .598; Both (+CSA) > Both (no CSA), *p* = .015*, *d* = .706																			
Too aggressive	2.22	1.02	2.43	1.27	2.12	0.99	2.67	0.86	1.89	0.77	2.13	0.81	1.78	0.79	1.93	0.76	3.294	.002*	.047	.444
	No significant differences detected between groups in follow-up analysis																			
Too open	1.89	0.96	2.27	0.95	2.33	0.66	2.46	0.46	2.16	0.71	2.31	0.84	2.06	0.75	2.35	0.64	1.654	.118	.024	.314

**Table 4.** Study 2: UPPS scores between exploratory groups.

	Group Means																			
	Victimization Only				Perpetration Only				Both				Non-Violence-Exposed Controls							
	No CSA (*n* = 25)		+CSA (*n* = 11)		No CSA (*n* = 29)		+CSA (*n* = 6)		No CSA (*n* = 104)		+CSA (*n* = 35)		No CSA (*n* = 260)		+CSA (*n* = 15)					
UPPS Scale	*m*	*SD*	*m*	*SD*	*m*	*SD*	*m*	*SD*	*m*	*SD*	*m*	*SD*	*m*	*SD*	*m*	*SD*	*F* (7, 478)	*p*	η2	*d*
Total	103.58	13.95	112.91	22.59	111.00	13.58	106.33	11.86	108.47	12.25	106.60	11.42	102.14	13.66	105.93	13.68	4.065	<.001*	.058	.496
	Both (no CSA) > Control (no CSA), *p* < .001*, *d* = .488																			
Premeditation	19.79	4.16	22.09	6.41	22.04	6.33	18.17	6.34	21.08	5.27	20.60	5.32	20.27	5.38	23.20	5.58	1.397	.204	.021	.293
Urgency	28.83	7.09	32.46	10.05	31.38	7.13	33.17	4.17	30.04	7.32	31.49	5.90	27.29	7.74	27.20	6.93	3.770	<.001*	.054	.478
	Both (+CSA) > Control (no CSA), *p* = .011*, *d* = .611																			
Sensation Seeking	35.96	6.81	37.73	5.48	37.76	7.41	34.00	6.29	38.23	6.57	33.89	5.41	35.50	6.97	34.80	6.48	2.765	.008*	.040	.408
	Both (no CSA) > Control (no CSA), *p* = .018*, *d* = .402; Both (no CSA) > Both (+CSA), *p* = .007*, *d* = .721																			
Perseverance	19.00	4.29	20.64	5.01	19.83	4.97	21.00	8.15	19.13	4.18	20.74	4.10	19.08	4.79	20.73	4.74	1.062	.387	.016	.255

## Data Availability

The data that support the findings of this study are available from the corresponding author, Jasmine Skorheim, upon reasonable request.
