# Supplementary material for: Victimization and Perpetration of Sexual Violence in College Men: The Characteristics of Social Ecology, Shared Risk Factors and Their Implications for Prevention
Source: J Aggress Maltreat Trauma. Author manuscript; Available in PMC 2025 Sep 24. (PMC12456444; doi:10.1080/10926771.2025.2549743)
Supplement: Supp 1 [file NIHMS2111626-supplement-Supp_1.docx]

**Supplemental Materials for: “Victimization and Perpetration of Sexual Violence in College Men: The Impact of Social Ecology and Shared Risk Factors on Prevention”**

**Study 1**

**Study 1: Participants and Procedures**

In addition to the measures assessed in Study 1, participants completed several other measures as part of a larger parent study, including the following: positive and negative affect schedule (PANAS), labeling vignettes, perceived stress scales, the Acceptance and Action Questionnaire (AAQ-II), the Inventory of Interpersonal Problems (IIP-32), and the UPPS Impulsive Behavior Scale. Analyses examining experimental vignette condition and study measures found no impact of experimental condition on study variables, including reports of childhood sexual abuse (χ2(1, *N*=485)=0.865, *p*=.352, Cramer’s *V*=.042, *d*=.085), adult/adolescent victimization (χ2(1, *N*=485)=1.957, *p*=.162, Cramer’s *V*=.064, *d*=.127), perpetration (χ2(1, *N*=485)=0.273, *p*=.601, Cramer’s *V*=.024, *d*=.048), victimization knowledge (χ2(1, *N*=485)=0.035, *p*=.851, Cramer’s *V*=.009, *d*=.017), perpetration knowledge (χ2(1, *N*=485)=0.240, *p*=.624, Cramer’s *V*=.022, *d*=.045), and responsibility for violence (*t*(469)=1.557, *p*=.120, *d*=.144).

**Measures**

*Marlowe-Crowne Social Desirability Scale (SDS).* The SDS consists of 33 items to assess social desirability through a series of true or false statements that reflect desired behavior rather than the truth of one’s behavior (e.g., “before voting I thoroughly investigate the qualifications of all the candidates”) (Crowne & Marlowe, 1960). This scale has evidence of adequate internal consistency, with Cronbach’s α=0.710 in this sample. This measure is used to control for social desirability effects and self-report biases.

*Responsibility for Violence.* To assess attitudes regarding responsibility for violence and how that may relate to overlap, the eight item Failure to Take Intervention Responsibility subscale from Burn’s (2009) 16 item Bystander Intervention Barriers Scale was used. This subscale has evidence of strong internal consistency with Cronbach’s α=0.868 in this sample. This subscale assesses responsibility for violence by measuring diffusion of responsibility, worthiness of the victim, and the relationship of the bystander to the potential victim or perpetrator. Participants were presented with each item from the subscale (e.g., “If I saw someone I didn’t know was at risk for being sexually assaulted, I would leave it up to his/her friends to intervene,”) and rated on a seven point Likert type scale from strongly disagree to strongly agree.

**Study 1: Results**

***Social Desirability***

To test for the effects of social desirability, partial correlations controlling for SDS scores were compared to bivariate correlations with differences ranging from 0 to 0.096, suggesting that social desirability did not have a confounding effect on results (Tracey, 2016); therefore, unadjusted analyses are reported.

***Responsibility for Violence***

We hypothesized that those with perpetration histories (Perpetration Only and Both groups) would report feeling less responsibility for violence (Scully & Marolla, 1984; Wegner et al., 2015; Ybarra et al., 2023). Hypotheses were not supported, (*F*(3, 482)=1.641, *p*=.179, η2=.010, *d*=.201). There were also no significant differences between those with and without peer perpetration knowledge for responsibility for violence scores (*t*(469)=-1.617, *p*=.107, *d*=-.158).

***Descriptive Statistics: Time Periods of Adolescent/Adult Sexual Violence***

Which time period participants reported the violence exposure occurred in was examined to give clues to the timing of victimization vs. perpetration. Of those in the victimization only group, 49.0% (*n*=25) reported the victimization experience occurring since the age of 14 while 51.0% (*n*=26) of those in the victimization only group reported the victimization experience occurring in the past year. Of note, 33.3% (*n*=17) reported experiences of victimization occurring both since the age of 14 and in the past year. Of those in the perpetration only group, 69.0% (*n*=20) reported the perpetration experience occurring since the age of 14 while 65.5% (*n*=19) of those in the perpetration only group reported the perpetration experience occurring in the past year. Of note, 41.4% (*n*=12) reported perpetration occurring both since the age of 14 and in the past year. Of those in the both group, 71.7% (*n*=104) reported experiencing victimization since the age of 14 while 73.1% (*n*=106) reported perpetrating since the age of 14. Furthermore, 84.1% (*n*=122) reported experiencing victimization in the past year and 86.2% (*n*=125) reported perpetration in the past year suggesting that at least for past year violence, incidents are occurring within short time-periods of one another. Of note, 60.7% (*n*=88) reported experiences of victimization both since the age of 14 and in the past year and 60.7% (*n*=88) reported perpetration both since the age of 14 and in the past year.

***Descriptive Statistics: CSA prevalence***

The prevalence rate of CSA in the sample was 13.8% (*n*=67). Of those in the victimization only group, 51.0% (*n*=26) reported CSA and 49.0% (*n*=25) reported adolescent/adult victimization. Of those in the both group, 28.3% (*n*=41) reported CSA and 71.1% (*n*=104) reported adolescent/adult victimization.

**Study 2**

**Study 2: Method**

***Participants and Procedures***

In addition to the measures assessed in Study 1, analyses examining experimental vignette condition and study measures found no impact of experimental condition on study variables, including IIP-32 scores (*t*(469)=-0.703, *p*=.482, *d*=-.065) and UPPS scores (*t*(469)=-0.568, *p*=.570, *d*=-.053).

**Study 2: Results**

***Descriptive Statistics***

Considering the victimization only group (*n*=36), 30.56% (*n*=11) reported victimization plus CSA. Considering the perpetration only group (*n*=35), 17.14% (*n*=6) reported perpetration plus CSA. Considering the both group (*n*=139), 25.18% (*n*=35) reported CSA. In the control group (*n*=275), 5.45% (*n*=15) reported CSA. A significantly greater proportion of the victimization only group (χ2=26.179, *p*<0.001*, Cramer’s *V*=0.290, *d*=0.478), the perpetration only group (χ2=6.717, *p*=0.010*, Cramer’s *V*=0.147, *d*=0.298), and the both group (χ2=33.832, *p*<0.001, Cramer’s *V*=0.286, *d*=0.597) reported CSA than those in the control group. Thus, most college men in this sample who reported CSA experienced sexual victimization again later in life (68.7%).

**References**

Crowne, D. P., & Marlowe, D. (1960). A new scale of social desirability independent of psychopathology. *Journal of Consulting Psychology, 24*(4), 349–354. https://doi.org/10.1037/h0047358

Burn, S.M. (2009). A situational model of sexual assault prevention through bystander intervention. *Sex Roles, 60*, 779–792. <https://doi.org/10.1007/s11199-008-9581-5>

Scully, D. & Marolla, J. (1984). Convicted rapists’ vocabulary of motive: Excuses and justifications. *Social Problems, 31*(5), 530-544. <https://doi.org/10.2307/800239>

Tracey, T. J. G. (2016). A note on socially desirable responding. *Journal of Counseling Psychology, 63*(2), 224–232. <https://doi-org.ezproxy.library.und.edu/10.1037/cou0000135>

Wegner, R., Abbey, A., Pierce, J., Pegram, S. E., & Woerner, J. (2015). Sexual assault perpetrators’ justifications for their actions: Relationships to rape supportive attitudes, incident characteristics, and future perpetration. *Violence Against Women, 21*(8), 1018–1037. <https://doi.org/10.1177/1077801215589380>

Ybarra, M. L., Strøem, I. F., Goodman, K. L., & Mitchell, K. J. (2023). Event characteristics of sexual violence perpetration against romantic partners versus non-romantic partners. *Journal of Interpersonal Violence, 38*(17–18), 10282–10308. <https://doi.org/10.1177/08862605231171411>

**Appendix A**

**Study 1: Victimization Knowledge Questionnaire**

1. In these questions "anyone" means any women you know, including your acquaintances, girlfriends, friends, and girl or women relatives. Do you know anyone who ever: - has given in to sex play (fondling, kissing, or petting but not intercourse) when she didn't want to because she was overwhelmed by a man's continual arguments and pressure?
   1. Yes
      1. You answered yes to do you know anyone who has ever: [item endorsed yes] What is your relationship to the woman who experienced this?
         1. Relative
         2. Friend
         3. Romantic Partner (girlfriend)
         4. Acquaintance
   2. No
2. In these questions "anyone" means any women you know, including your acquaintances, girlfriends, friends, and girl or women relatives. Do you know anyone who ever: - has had sex play (fondling, kissing, or petting but not intercourse) when she didn't want to because a man threatened or used some degree of physical force (twisting your arm, holding you down, etc.) to make her?
   1. Yes
      1. You answered yes to do you know anyone who has ever: [item endorsed yes] What is your relationship to the woman who experienced this?
         1. Relative
         2. Friend
         3. Romantic Partner (girlfriend)
         4. Acquaintance
   2. No
3. In these questions "anyone" means any women you know, including your acquaintances, girlfriends, friends, and girl or women relatives. Do you know anyone who ever: - had a man attempt sexual intercourse (get on top of her, attempt to insert his penis) when she didn't want to by threatening or using some degree of force (twisting her arm, holding her down, etc.) but intercourse did not occur?
   1. Yes
      1. You answered yes to do you know anyone who has ever: [item endorsed yes] What is your relationship to the woman who experienced this?
         1. Relative
         2. Friend
         3. Romantic Partner (girlfriend)
         4. Acquaintance
   2. No
4. In these questions "anyone" means any women you know, including your acquaintances, girlfriends, friends, and girl or women relatives. Do you know anyone who ever: - had a man attempt sexual intercourse (get on top of her, attempt to insert his penis) when she didn't want to by giving her alcohol or drugs, but intercourse did not occur?
   1. Yes
      1. You answered yes to do you know anyone who has ever: [item endorsed yes] What is your relationship to the woman who experienced this?
         1. Relative
         2. Friend
         3. Romantic Partner (girlfriend)
         4. Acquaintance
   2. No
5. In these questions "anyone" means any women you know, including your acquaintances, girlfriends, friends, and girl or women relatives. Do you know anyone who ever: - gave in to sexual intercourse when she didn't want to because she was overwhelmed by a man's continual arguments and pressure?
   1. Yes
      1. You answered yes to do you know anyone who has ever: [item endorsed yes] What is your relationship to the woman who experienced this?
         1. Relative
         2. Friend
         3. Romantic Partner (girlfriend)
         4. Acquaintance
   2. No
6. In these questions "anyone" means any women you know, including your acquaintances, girlfriends, friends, and girl or women relatives. Do you know anyone who ever: - had a man force sexual intercourse (get on top of her, attempt to insert his penis) when she didn't want to by giving her alcohol or drugs?
   1. Yes
      1. You answered yes to do you know anyone who has ever: [item endorsed yes] What is your relationship to the woman who experienced this?
         1. Relative
         2. Friend
         3. Romantic Partner (girlfriend)
         4. Acquaintance
   2. No
7. In these questions "anyone" means any women you know, including your acquaintances, girlfriends, friends, and girl or women relatives. Do you know anyone who ever: - had a man force sexual intercourse (get on top of her, attempt to insert his penis) when she didn't want to by threatening or using some degree of force (twisting her arm, holding her down, etc.)?
   1. Yes
      1. You answered yes to do you know anyone who has ever: [item endorsed yes] What is your relationship to the woman who experienced this?
         1. Relative
         2. Friend
         3. Romantic Partner (girlfriend)
         4. Acquaintance
   2. No
8. In these questions "anyone" means any women you know, including your acquaintances, girlfriends, friends, and girl or women relatives. Do you know anyone who ever: - had sex acts (anal or oral intercourse or penetration by objects other than a penis) when she didn't want to because a man threatened or used some degree of physical force (twisting her arm, holding her down, etc.) to make her?
   1. Yes
      1. You answered yes to do you know anyone who has ever: [item endorsed yes] What is your relationship to the woman who experienced this?
         1. Relative
         2. Friend
         3. Romantic Partner (girlfriend)
         4. Acquaintance
   2. No

**Appendix B**

**Study 1: Perpetration Knowledge Questionnaire**

1. In these questions "anyone" means any men you know, including your male acquaintances, friends, and boy or male relatives. Do you know anyone who ever: - used continual arguments and pressure to get a girl to give in to sex play (fondling, kissing, or petting, but not intercourse) when she didn't want to?
   1. Yes
      1. You answered yes to do you know anyone who has ever: [item endorsed yes] What is your relationship to the woman who experienced this?
         1. Relative
         2. Friend
         3. Romantic Partner
         4. Acquaintance
   2. No
2. In these questions "anyone" means any men you know, including your male acquaintances, friends, and boy or male relatives. Do you know anyone who ever: - threatened or used some degree of physical force (twisting her arm, holding her down, etc.) to make a woman have sex play (fondling, kissing, or petting, but not intercourse) when she didn't want to?
   1. Yes
      1. You answered yes to do you know anyone who has ever: [item endorsed yes] What is your relationship to the woman who experienced this?
         1. Relative
         2. Friend
         3. Romantic Partner
         4. Acquaintance
   2. No
3. In these questions "anyone" means any men you know, including your male acquaintances, friends, and boy or male relatives. Do you know anyone who ever: - threatened or used some degree of physical force (twisting her arm, holding her down, etc.) to attempt sexual intercourse with a woman (get on top of her, attempt to insert his penis) when she didn't want to but intercourse did not occur?
   1. Yes
      1. You answered yes to do you know anyone who has ever: [item endorsed yes] What is your relationship to the woman who experienced this?
         1. Relative
         2. Friend
         3. Romantic Partner
         4. Acquaintance
   2. No
4. In these questions "anyone" means any men you know, including your male acquaintances, friends, and boy or male relatives. Do you know anyone who ever: - attempted sexual intercourse with a woman (get on top of her, attempt to insert his penis) when she didn't want to by giving her alcohol or drugs, but intercourse did not occur?
   1. Yes
      1. You answered yes to do you know anyone who has ever: [item endorsed yes] What is your relationship to the woman who experienced this?
         1. Relative
         2. Friend
         3. Romantic Partner
         4. Acquaintance
   2. No
5. In these questions "anyone" means any men you know, including your male acquaintances, friends, and boy or male relatives. Do you know anyone who ever: - used continual arguments and pressure to get a woman to give in to sexual intercourse when she didn't want to?
   1. Yes
      1. You answered yes to do you know anyone who has ever: [item endorsed yes] What is your relationship to the woman who experienced this?
         1. Relative
         2. Friend
         3. Romantic Partner
         4. Acquaintance
   2. No
6. In these questions "anyone" means any men you know, including your male acquaintances, friends, and boy or male relatives. Do you know anyone who ever: - has had sexual intercourse with a woman when she didn't want to by giving her alcohol or drugs?
   1. Yes
      1. You answered yes to do you know anyone who has ever: [item endorsed yes] What is your relationship to the woman who experienced this?
         1. Relative
         2. Friend
         3. Romantic Partner
         4. Acquaintance
   2. No
7. In these questions "anyone" means any men you know, including your male acquaintances, friends, and boy or male relatives. Do you know anyone who ever: - has sexual intercourse with a woman when she didn't want to because he threatened or used some degree of force (twisting her arm, holding her down, etc.) to make her?
   1. Yes
      1. You answered yes to do you know anyone who has ever: [item endorsed yes] What is your relationship to the woman who experienced this?
         1. Relative
         2. Friend
         3. Romantic Partner
         4. Acquaintance
   2. No
8. In these questions "anyone" means any men you know, including your male acquaintances, friends, and boy or male relatives. Do you know anyone who ever: - had sex acts with a woman (anal or oral intercourse or penetration by objects other than the penis) when she didn't want to because he threatened or used some degree or physical force (twisting her arm, holding her down, etc.) to make her?
   1. Yes
      1. You answered yes to do you know anyone who has ever: [item endorsed yes] What is your relationship to the woman who experienced this?
         1. Relative
         2. Friend
         3. Romantic Partner
         4. Acquaintance
   2. No

**Appendix C**

| **Relationship Characteristic** | **Group** | | | | | | | | **χ2 (3, *N*=485)** | ***p*** | **Cramer’s V** | ***d*** |
| --- | --- | --- | --- | --- | --- | --- | --- | --- | --- | --- | --- | --- |
|  | **Victimization Only**  **(*n*=51)** | | **Perpetration Only**  **(*n*=29)** | | **Both**  **(*n*=145)** | | **Nonviolence Exposed Controls**  **(*n*=260)** | |  |  |  |  |
| Has a close friend | 86.0% | | 93.1% | | 88.2% | | 90.7% | | 1.693 | .639 | .060 | .118 |
| More than one close friend | 82.0% | | 86.2% | | 84.7% | | 85.4% | | 0.423 | .935 | .030 | .059 |
| Gender of best friend(s) | Male 36.0% | | Male 44.8% | | Male 38.5% | | Male 32.9% | | 3.429 | .330 | .084 | .169 |
|  | Female 16.0% | | Female 6.9% | | Female 7% | | Female 8.9% | | 3.880 | .275 | .089 | .180 |
|  | Both 48.0% | | Both 48.3% | | Both 54.5% | | Both 58.1% | | 1.407 | .704 | .054 | .108 |
| Friends that are not the same sexual orientation | Yes 54.0% | | Yes 37.9% | | Yes 47.9% | | Yes 44.1% | | 3.236 | .357 | .082 | .164 |
|  | Maybe 18.0% | | Maybe 13.8% | | Maybe 13.2% | | Maybe 16.2% | | 0.750 | .861 | .039 | .079 |
|  | No 28.0% | | No 48.3% | | No 38.9% | | No 39.7% | | 3.715 | .294 | .088 | .176 |
| **Friends** | **Group Means** | | | | | | | | ***F* (3, 482)** | ***p*** | **η2** | ***d*** |
|  | **Victimization Only**  **(n=51)** | | **Perpetration Only**  **(n=29)** | | **Both**  **(n=145)** | | **Nonviolence Exposed Controls**  **(n=260)** | |  |  |  |  |
|  | *M* | *SD* | *M* | *SD* | *M* | *SD* | *M* | *SD* |  |  |  |  |
| Men | 5.34 | 5.80 | 4.46 | 3.54 | 4.82 | 4.97 | 4.18 | 4.63 | 0.947 | .418 | .007 | .168 |
| Women | 3.37 | 3.98 | 2.24 | 1.58 | 2.48 | 1.92 | 2.35 | 2.40 | 2.193 | .088 | .016 | .255 |
| Total | 8.71 | 8.32 | 6.70 | 4.32 | 7.28 | 6.00 | 6.53 | 6.25 | 1.550 | .201 | .011 | .211 |

Appendix C. Study 1: Relationship Characteristics Between Group
